# Perforation of the colon in response to the blast effect of an extraperitoneal gunshot injury

**DOI:** 10.1016/j.ijscr.2023.109051

**Published:** 2023-11-12

**Authors:** Mohammad Sina Salimikoochi, Yousef Soleimani

**Affiliations:** Educational and therapeutic hospital of Golestan hospital, Ahvaz Jundishapur University of Medical Sciences, Ahvaz, Iran

**Keywords:** Blast effect, Gunshot, Colon perforation, Trauma

## Abstract

**Introduction and importance:**

Gunshot injury has many medical aspects, and the blast effect is a rare one that needs a precise first emergency examination.

**Case presentation:**

A 16-year-old male who was injured by a gunshot. Upon arrival at the emergency department, he was treated as a trauma call. There was only one bullet that entered from the outer edge of the right ASIS and exited inside the right buttock approximately 10 cm lower tangentially and no bullet entered the abdomen. Initial assessments and imaging revealed no underlying pathology findings, and the patient's vital signs were stable. Epigastric and RUQ tenderness and CT findings prompted us to perform a laparotomy. During laparotomy, we discovered a perforation in the hepatic flexure of the colon.

**Clinical discussion:**

It is believed that as the peritoneal cavity is not touched by bullet, the colon is perforated because of the blast effect of the gunshot. Although blast effects from gunshots or shotguns are rare, especially in intact abdomens, clinical staff should keep this effect in mind. This will ensure that they don't misinterpret the bullet path and external torso and don't miss anything subtle during their initial clinical examination.

**Conclusions:**

This case highlights the possibility of blast effect in gunshot cases and injury to organs that aren't in the bullet's path. Laparotomy is recommended if CT scan shows thickened colon wall in patients with extraperitoneal gunshot injury.

## Highlights

• A 16-year-old male who was injured by a gunshot and initial assessment revealed no underlying pathology findings.
• Epigastric and RUQ tenderness prompted us to perform a laparotomy.
• the peritoneal cavity is not touched and the colon is perforated because of the blast effect of the gunshot.

## 1 Introduction

The blast effect is blunt and comes from a physical wave and does not need direct contact to do damage. In contrast to bombs and explosions, the blast effect from penetrating wounds like gunshot wounds is scarce. The blast effect can result in blunt injury to the entire abdomen [1,2].

Considering the increasing crimes involving gun crime worldwide, it is more likely to face a case like this.

Clinical staff and surgeons should be aware of the blast effect and be more subtle in their initial examination. Delayed or missed diagnosis is associated with high morbidity [2,3].

Herein We report a case with perforation of ascending colon caused by an extraperitoneal gunshot wound.

## 2 Case presentation

**Fig. 1:**
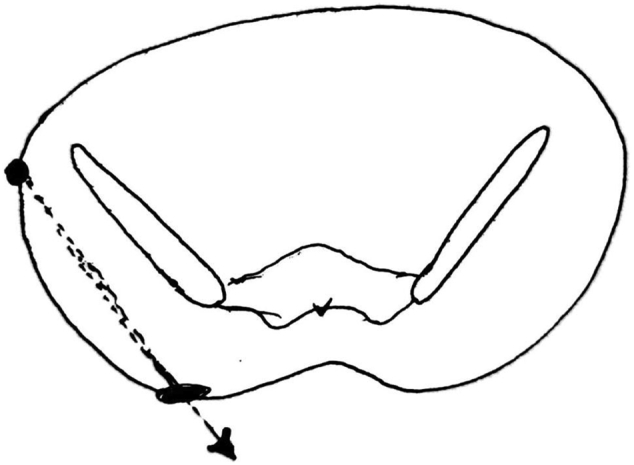
Pathway of the bullet between entry and exit wounds in the body.

**Fig. 2:**
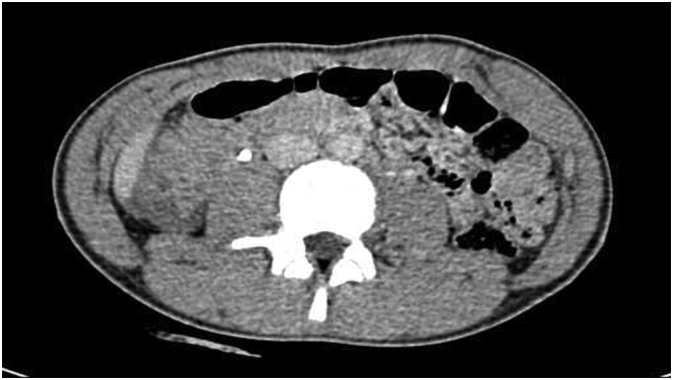
CT scan of the abdomen, showing just a little thickening of the colon wall and no significant findings.

**Fig. 3:**
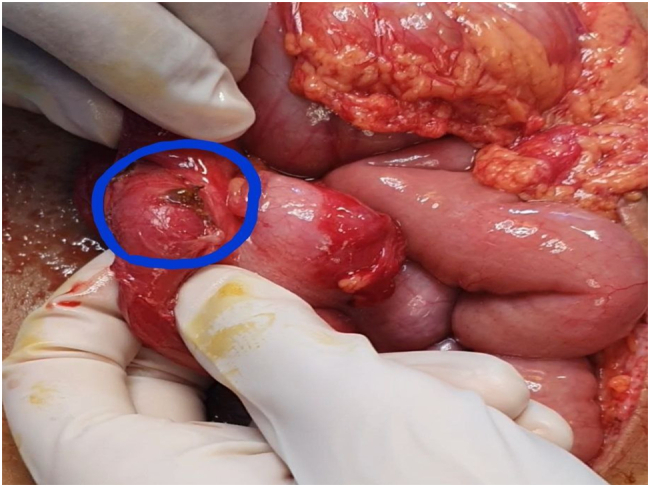
Perforation of the hepatic flexure of the colon.

A 16-year-old male patient was hit by a bullet in the outer edges of the right anterior superior iliac spine (ASIS), where the exit point was about 10 cm lower and more inside the right buttock (see Fig. 1). The patient's vital signs were stable at the time of the visit, and after performing primary and secondary measures, focused assessment with sonography in trauma (FAST) was also performed, the result of which was negative. No wounds or evidence of other injuries were observed in the examinations. In the abdominal examination, the only positive finding was mild tenderness in the right upper quadrant (RUQ). Rectal examination was normal and no evidence of bleeding or damage to the rectum and anus was observed. In the initial tests, the only significant finding was the presence of leukocytosis in 17,000. There was no active bleeding from the patient's wound, and no connection with the abdominal cavity or pelvis was seen in the examination of the wound. The pulses of the upper and lower extremities were full and regular. Neurological examination was normal. There was no hematuria. Due to the stability of the patient's vital signs, a CT scan was performed instead of an abdomen X-ray. In the CT scan we could detect slight isodensity area between liver and hepatic flexure of colon suggesting a slight amount of ascites. Another notable finding in the CT scan was a small thickening of the colon (see Fig. 2). Finally, as the severity of colon injury in the CT scan wasn't clear and the tenderness was spreading from RUQ to epigastric we performed laparotomy. The abdomen was opened with a midline incision, the abdominal cavity was clean, and no blood or digestive secretions were seen. The stomach and small intestine were examined from the ligament of Treitz to the end. No pathological findings were seen. The colon was also examined. A perforation of less than one centimeter was seen on the posterior surface of the hepatic flexure of the colon, which was sealed by the surrounding soft tissue (see Fig. 3). Due to the cleanliness of the abdominal cavity, the initial repair of the colon was performed and after washing, the abdomen was dried and closed. The bullet wound was also washed and debrided. Finally, after 6 days, the patient was discharged with a good general condition.

## 3 Methods

The work has been reported in line with the SCARE criteria [4].

## 4 Discussion

Blast effect is the impression of blast wave. As this wave is a mechanical wave and transfers force and energy.it can transfer energy to other subject thus can cause damage Recent studies report more frequent instances of intraperitoneal damage caused by blast effects.

blast-related injury can be classified into four main categories: primary (sudden increase in air pressure), secondary (missiles energized by the blast), tertiary (displacement of the victim by the blast wind), and quaternary (flashy burns, asphyxia) [2,5].

It is also more common for the colon and less often the small intestine to be affected by primary blast injury.

Explosive devices such as bombs and other explosive devices are frequently the cause of blast injuries. In spite of this, even tangential gunshot or shotgun injuries can damage abdominal organs through kinetic energy transmission [6]. An immediate rupture of the colon can result from the blast effect (primary perforation) or it can be delayed (secondary perforation). A secondary perforation is preceded by tissue disruption, hemorrhage, hematoma, inflammatory cell infiltration, and necrosis, which are all combined to cause gangrene.

Although blast effects from gunshots or shotguns are rare, clinical staff should keep this effect in mind. This will ensure that they don't misinterpret the bullet path and external torso and don't miss anything subtle during their initial clinical examination.

Blunt intestinal trauma is relatively uncommon and can be a diagnostic challenge with a potentially life-threatening outcome. For example, in our case, There was no air in the CT scan and we detected just a small thickening of the colon wall and a slight isodensity area between the liver and hepatic flexure of the colon.

In some cases, perforation of the colon may occur within 72 h after the trauma [7]. Therefore, patients exposed to significant explosive blasts should be monitored carefully for at least 48 to 72 h [7,8], especially those with other injuries who require analgesics. It may also be necessary to perform pertinent diagnostic tests, such as CT scans, sonography, diagnostic peritoneal lavage, laparoscopy, or even a laparotomy, in addition to careful clinical observation. A laparotomy was performed on our patient based on tenderness, leukocytosis, CT findings, and positive clinical findings for peritonitis. Diagnostic laparoscopy can be performed when the intraperitoneal involvement is unclear. This is in patients who have sustained blunt or penetrating trauma but have stable hemodynamics and no evidence of intraperitoneal injury.

## 5 Conclusion

As a result of this case, laparotomy should be considered if the colon wall is thickened on CT scan in the patient of an extraperitoneal gunshot injury. It is important to keep in mind that gunshots can cause a blast effect. This can lead to injuries to organs that aren't in the bullet's path.

## Abbreviations

ASIS: anterior superior iliac spine
RUQ: right upper quadrant
FAST: focused assessment with sonography in trauma

## Ethical approval

Not applicable.

## Funding

The authors declare that they have no funding resources.

## CRediT authorship contribution statement

Mohammad Sina Salimikoochi conceptualized the report and performed the surgery and Yousef Soleimani interpreted and wrote the manuscript.

Both authors have approved the submitted version and have agreed to be personally accountable for the authors' contributions.

## Consent for publication

Written informed consent was obtained from the patient for publication of this case report and any accompanying images. A copy of the written consent is available for review by the Editor-in-Chief of this journal.

## Guarantor

Yousef Soleimani.

## Declaration of competing interest

The authors declare that they have no competing interests.

## Data Availability

Not applicable.
